# Determinants of plasma levels of proglucagon and the metabolic impact of glucagon receptor signalling: a UK Biobank study

**DOI:** 10.1007/s00125-024-06160-1

**Published:** 2024-05-06

**Authors:** Marie Winther-Sørensen, Sara L. Garcia, Andreas Bartholdy, Maud E. Ottenheijm, Karina Banasik, Søren Brunak, Charlotte M. Sørensen, Lise Lotte Gluud, Filip K. Knop, Jens J. Holst, Mette M. Rosenkilde, Majken K. Jensen, Nicolai J. Wewer Albrechtsen

**Affiliations:** 1 Department for Clinical Biochemistry, Copenhagen University Hospital – Bispebjerg and Frederiksberg, University of Copenhagen, Copenhagen, Denmark; 2 Novo Nordisk Foundation Center for Protein Research, Faculty of Health and Medical Sciences, University of Copenhagen, Copenhagen, Denmark; 3 Department of Biomedical Sciences, Faculty of Health and Medical Sciences, University of Copenhagen, Copenhagen, Denmark; 4 Section of Epidemiology, Department of Public Health, Faculty of Health and Medical Sciences, University of Copenhagen, Copenhagen, Denmark; 5 Gastro Unit, Copenhagen University Hospital – Hvidovre, Hvidovre, Denmark; 6 Department of Clinical Medicine, Faculty of Health and Medical Sciences, University of Copenhagen, Copenhagen, Denmark; 7 Steno Diabetes Center Copenhagen, Herlev, Denmark; 8 Center for Clinical Metabolic Research, Gentofte Hospital, University of Copenhagen, Hellerup, Denmark; 9 Novo Nordisk Foundation Center for Basic Metabolic Research, Faculty of Health and Medical Sciences, University of Copenhagen, Copenhagen, Denmark

**Keywords:** Glucagon receptor, MASLD, Obesity, Proglucagon, Type 2 diabetes, UK Biobank

## Abstract

**Aims/hypotheses:**

Glucagon and glucagon-like peptide-1 (GLP-1) are derived from the same precursor; proglucagon, and dual agonists of their receptors are currently being explored for the treatment of obesity and metabolic dysfunction-associated steatotic liver disease (MASLD). Elevated levels of endogenous glucagon (hyperglucagonaemia) have been linked with hyperglycaemia in individuals with type 2 diabetes but are also observed in individuals with obesity and MASLD. GLP-1 levels have been reported to be largely unaffected or even reduced in similar conditions. We investigated potential determinants of plasma proglucagon and associations of glucagon receptor signalling with metabolic diseases based on data from the UK Biobank.

**Methods:**

We used exome sequencing data from the UK Biobank for ~410,000 white participants to identify glucagon receptor variants and grouped them based on their known or predicted signalling. Data on plasma levels of proglucagon estimated using Olink technology were available for a subset of the cohort (~40,000). We determined associations of glucagon receptor variants and proglucagon with BMI, type 2 diabetes and liver fat (quantified by liver MRI) and performed survival analyses to investigate if elevated proglucagon predicts type 2 diabetes development.

**Results:**

Obesity, MASLD and type 2 diabetes were associated with elevated plasma levels of proglucagon independently of each other. Baseline proglucagon levels were associated with the risk of type 2 diabetes development over a 14 year follow-up period (HR 1.13; 95% CI 1.09, 1.17; *n*=1562; *p*=1.3×10^−12^). This association was of the same magnitude across strata of BMI. Carriers of glucagon receptor variants with reduced cAMP signalling had elevated levels of proglucagon (β 0.847; 95% CI 0.04, 1.66; *n*=17; *p*=0.04), and carriers of variants with a predicted frameshift mutation had higher levels of liver fat compared with the wild-type reference group (β 0.504; 95% CI 0.03, 0.98; *n*=11; *p*=0.04).

**Conclusions/interpretation:**

Our findings support the suggestion that glucagon receptor signalling is involved in MASLD, that plasma levels of proglucagon are linked to the risk of type 2 diabetes development, and that proglucagon levels are influenced by genetic variation in the glucagon receptor, obesity, type 2 diabetes and MASLD. Determining the molecular signalling pathways downstream of glucagon receptor activation may guide the development of biased GLP-1/glucagon co-agonist with improved metabolic benefits.

**Data availability:**

All coding is available through https://github.com/nicwin98/UK-Biobank-GCG

**Graphical Abstract:**

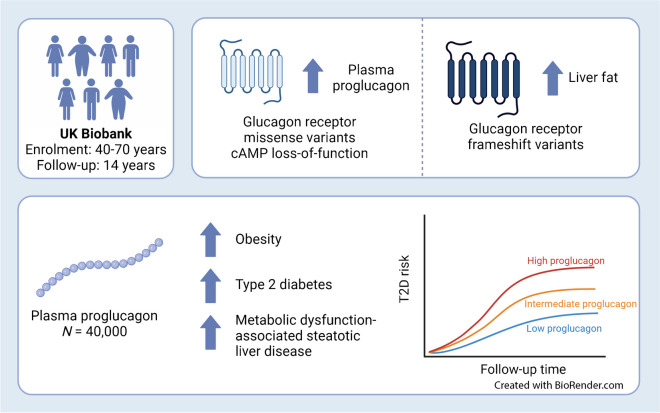

**Supplementary Information:**

The online version of this article (10.1007/s00125-024-06160-1) contains peer-reviewed but unedited supplementary material.


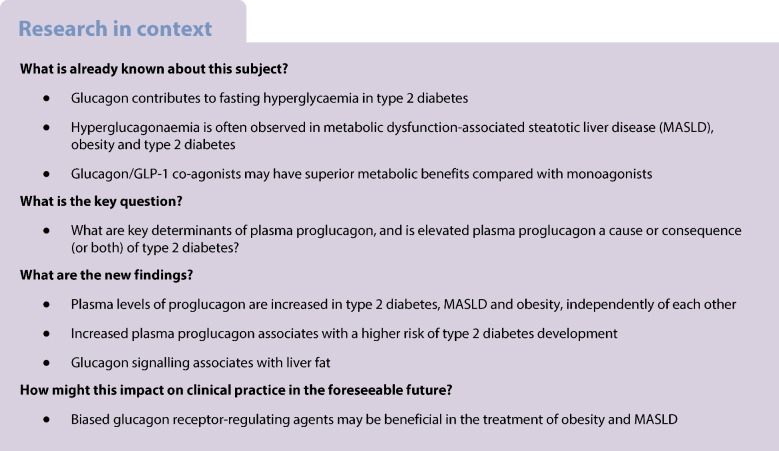


## Introduction

The proglucagon gene (*GCG*) encodes several circulating peptides/hormones including glucagon, glucagon-like peptide-1 (GLP-1), glucagon-like peptide 2, glicentin, oxyntomodulin, and major proglucagon fragment (MPGF). Glucagon and GLP-1 impact on glucose control, food intake, and hepatic protein and lipid metabolism [1], and co-agonists of these two hormones are being tested in clinical trials for the treatment of obesity and metabolic dysfunction-associated steatotic liver disease (MASLD) [2, 3]. Glucagon binds to and acts via the glucagon receptor, belonging to class B1 of the superfamily of G protein-coupled receptors, signalling through Gα_s_ (stimulating the adenylate cyclase/cAMP/protein kinase A pathway) and Gα_q_ (signalling through the phospholipase C [PLC]/inositol phosphate [IP3]/calcium/calmodulin pathway). Like other class B1 receptors, the glucagon receptor recruits β-arrestin, which sterically alters the binding between the receptor and the G protein and regulates internalisation [4]. The molecular pharmacological phenotypes of 38 missense variants of the glucagon receptor were recently described at the level of cAMP signalling and β-arrestin recruitment [5], whereas similar systematic investigations at the level of the PLC/IP3 pathway are still lacking.

Increased plasma levels of glucagon (hyperglucagonaemia) are associated with fasting hyperglycaemia in people with type 2 diabetes but are also observed in individuals with obesity and/or MASLD [6–8]. An important gap in the understanding of the pathophysiological role of glucagon in metabolic diseases lies in elucidating whether increased plasma levels of glucagon result from: (1) obesity, (2) MASLD, (3) type 2 diabetes, or (4) a combination of these. A key question in this context is whether hyperglucagonaemia is merely an epiphenomenon of dysmetabolic conditions or a direct contributor to the development of type 2 diabetes. Elucidation of this has been challenged due to lack of sufficient matching of body weight, age, sex, MASLD status and kidney function, in particular because of limited sample size across reported clinical studies.

To determine the role of glucagon and glucagon-related peptides in type 2 diabetes, and whether hyperproglucagonaemia exists independently of obesity, MASLD and type 2 diabetes, we analysed data from the UK Biobank including data from nearly 500,000 individuals. The dataset included plasma proglucagon data for ~40,000 individuals, ~15 years follow-up data on incident type 2 diabetes development, amino acid quantification for ~230,000 individuals, liver fat quantification for ~35,000 individuals, and exome sequencing, allowing investigations of the potential impact of glucagon receptor variants on clinical features.

## Materials and methods

### The UK Biobank

The UK Biobank is a large prospective research resource including half a million participants aged 40 to 69 at the time of inclusion from the UK. The biobank encompasses genetic, lifestyle and health data derived from various sources such as questionnaires, physical assessments, biological specimens, imaging and the continual monitoring of health-related outcomes, as described in detail previously [9]. Participants who withdrew from the biobank were excluded from all analyses (*n*=179, updated 14 November 2023). We excluded individuals who were not categorised as white and individuals with sex chromosome aneuploidy. A list of field names used in the study is available in electronic supplementary material (ESM) Table 1.

### Proteomics data processing

Proteomics data for 1464 proteins and 54,219 individuals were generated by Olink (Uppsala, Sweden) using the Olink Explore 1536 PEA (proximity extension assay). The proteomics measurements are available as Normalised Protein eXpression (NPX) units (an arbitrary and normalised unit on a log_2_ scale). The data was accessed through the DNAnexus platform (DNAnexus, Mountain View, CA, USA) and processed locally. Processing of the raw data and normalisation has been described elsewhere [10, 11]. Overall, there was 2.9% missing data (4.5% missing data for proglucagon). In preliminary analyses, we found no association between the proportion of missing data and sex or having a glucagon receptor variant compared with the wild-type (see Methods: Exome sequencing analysis and variant annotation). We filtered out individuals and proteins with >10% missing data (4 proteins and 3298 individuals were excluded). On the basis of the assumption that missing values are missing because they are below the limit of detection, we used the MinProb method (ImputeLCMD package for R, v. 2.0; R Foundation, Vienna, Austria) to impute the remaining 1.1% missing data (1.7% missing data for glucagon). This approach aims to preserve the overall structure of the dataset by conservatively estimating the missing values; however, imputation may lead to a loss of information, particularly in terms of variability. The resulting proteomics sub-cohort consisted of 40,164 individuals, and differential expression analysis was conducted on 1460 proteins to uncover plasma proteins potentially regulated by glucagon receptor signalling. In a prospective analysis the association between concentrations of proglucagon and the risk of incident diabetes was evaluated.

### Exome sequencing analysis and variant annotation

We analysed the whole-exome sequencing data of 469,914 individuals from the UK Biobank [9]. The UK Biobank whole-exome sequencing data was reference-aligned with the Original Quality Functional Equivalent protocol previously described [12]. This protocol uses Burrows–Wheeler Alignment Maximal Exact Matches (BWA-MEM) [13] to map all the reads to the human reference genome GRCh38 [14]. Variant calling was performed using DeepVariant (v.1.5.0) [15]. We filtered the genomic Variant Call Format (gVCF) files for each sample, restricted to the location of the glucagon receptor at chr17: 81,804,150 to 81,814,008 forward strand. The analyses were conducted on the Research Analysis Platform (https://ukbiobank.dnanexus.com, accessed August 2023). A computing instance equipped with 36 CPU threads was chosen to run 36 bcftools (Swiss army knife v.4.9.1; DNAnexus, Mountain View, CA, USA) parallel jobs and automatically launch new jobs when the previous jobs had finished. The output csv files were subsequently merged using Python. The scripts used to filter the gVCF files from UK Biobank and merge the output csv files are available at https://github.com/nicwin98/UK-Biobank-GCG.

We filtered for genotype quality (GQ) <20, depth (DP) <10, and allele balance (AB, for the minor allele) <0.2. The genetic variants were annotated for their sequence effect with opencravat.org (accessed August 2023) [16]. We created a variant group ‘Frameshift’ for predicted loss-of-function (LoF) alleles. This group included the sequence ontologies frameshift elongation, frameshift truncation, in-frame deletion, in-frame insertion, start lost, and stop gained (see ESM Table 2). Missense variants were denoted as the reference amino acid (1 letter code), the amino acid position, followed by the alternative amino acid. Missense variants were subsequently categorised as G40S heterozygotes and G40S homozygotes (pooled in the proteomics sub-cohort), or as ‘cAMP LoF’ based on a previous study reporting the molecular phenotype of 38 missense variants [5].

### Incident type 2 diabetes and survival analyses

We defined type 2 diabetes based on hospital diagnoses encoded as E11 or E14 in the ICD-10 classification system (https://icd.who.int/browse10/2019/en). We excluded individuals with a diagnosis of type 1 diabetes (E10). Prevalent cases were defined as: (1) probable and possible type 2 diabetes based on the Eastwood algorithm [17], (2) with a baseline HbA_1c_ greater than 48 mmol/mol (6.5%) (a recommended cutoff point for diagnosing type 2 diabetes), or (3) a diagnosis before or within 6 months after the enrolment visit. A list of fields used for the definition of diseases is available in ESM Table 3. After exclusion of 1833 prevalent cases, a total of 1562 developed incident type 2 diabetes during follow-up (median follow-up time: 14.75 years).

Risk time (in months) was defined from date of baseline examination (between 2006 and 2010) where the blood sample for proteomics analysis was obtained, to date of type 2 diabetes diagnosis, death, end of follow-up (defined as the last updated version of the hospital register [3 October 2021]), or loss to follow-up, whichever occurred first. Data on loss to follow-up was last updated in May 2017, and censored individuals were included in the analysis up to the point of censoring.

For the Kaplan–Meier survival analysis, the cohort was stratified into tertiles based on their plasma proglucagon levels at the baseline visit. Pairwise comparison using the Logrank test with Bonferroni correction for multiple testing was used to compare survival curves between the three subgroups. Using Cox proportional hazard regression with adjustment for age and sex, we explored BMI (continuously) as a potential intermediate. However, due to lacking model fit, our final analysis instead stratified on BMI categories (BMI <25 kg/m^2^; 25 kg/m^2 ^≤ BMI <30 kg/m^2^; BMI ≥30 kg/m^2^) and addressed whether the association between proglucagon and incident type 2 diabetes differed across BMI categories. Proportional hazards and linearity assumptions for all covariates were assessed with Schoenfeld and Martingale residual plots, respectively. Survival analyses were done using the Survival package for R (v3.5-4) [18].

### Statistical methods

For association analyses, we used the following variables: age when attending the assessment centre; sex; BMI; baseline type 2 diabetes (defined as probable and possible type 2 diabetes based on the Eastwood algorithm) [17]; liver fat (quantified by MRI–proton density fat-fraction [PDFF]) at the second repeat visit) [19]; weekly alcohol consumption; glucose; HbA_1c_; amino acids. BMI was used continuously or as a binary variable (non-obese, BMI <25 kg/m^2^; obese, BMI ≥30 kg/m^2^). Liver fat was used as a continuous variable or a binary variable to define MASLD (non-MASLD, <5.5%; MASLD, ≥5.5%), with individuals having a weekly alcohol intake above 17.5 units for women and 26.25 units for men being excluded in both cases. A list of type 2 diabetes medications tested for confounding is provided in ESM Table 4.

Differences in plasma levels of proglucagon by disease groups and respective control groups were assessed by unpaired *t* tests. For linear models, increments of independent variables were set to 5% for liver fat, 5 years for age, and 5 mmol/l for serum creatinine. BMI, glucose, HbA_1c_ and amino acids were normalised to the SD of each variable. Glucagon receptor variant groups were tested for association of binary traits with logistic regression with Firth correction.

Differential expression analysis of the wild-type reference group vs the glucagon receptor variant groups (i.e. frameshift, cAMP loss-of-function, and G40S) respectively, was conducted using Limma software (v.3.56.2) [20]. Protein NPX values from the Olink proteomics analysis were the outcome and variant group, age and sex were predictors. Multiple comparisons were adjusted using the Benjamini–Hochberg method. R version 4.3.0 was used for all analyses.

## Results

### Plasma proglucagon is elevated in obesity, type 2 diabetes and MASLD and associates with increased risk of incident type 2 diabetes

**Fig. 1:**
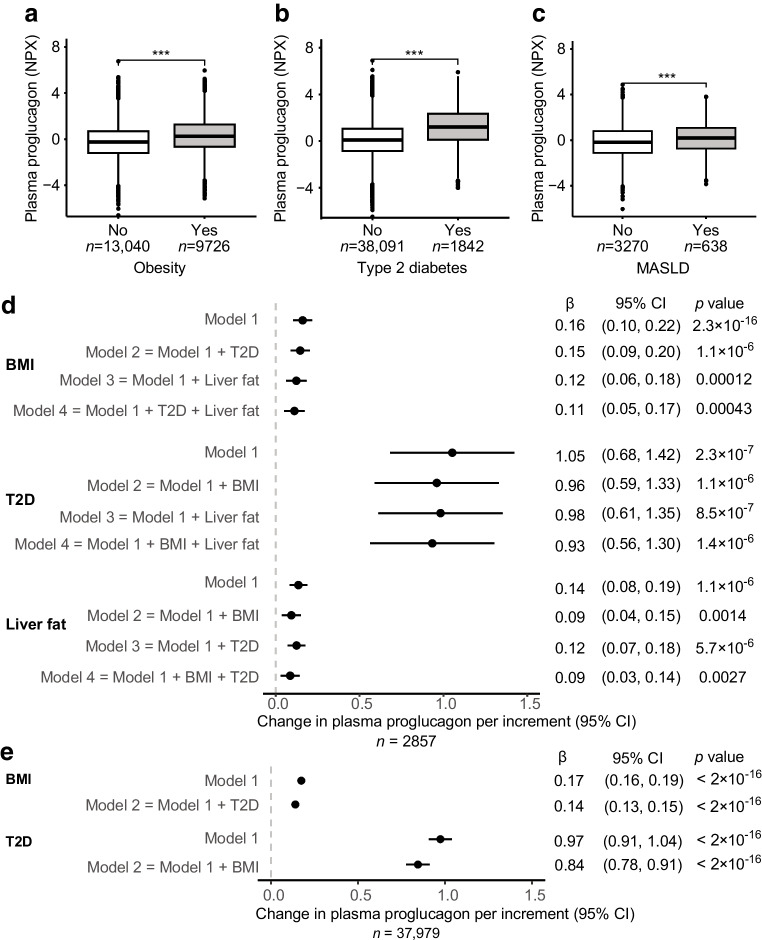
Plasma proglucagon is elevated in obesity, type 2 diabetes and MASLD. Plasma proglucagon in (**a**) lean individuals (BMI <25) and individuals with obesity (BMI ≥30), (**b**) individuals with and without type 2 diabetes, and (**c**) individuals with and without MASLD. The number of individuals in each group is shown. Data are shown as boxplots with quartiles and whiskers representing 1.5 times the interquartile range. Data points beyond the whiskers are plotted individually. Data is analysed by unpaired *t* test: ***p<0.001. (**d**) Multiple linear regression analyses were performed with plasma proglucagon from Olink proteomics as the dependent variable and BMI, T2D and per cent liver fat (quantified by PDFF) as independent variables. Increments are given as SDs of BMI and a 5% increase for liver fat. Model 1 for each variable included adjustment for age, sex, fasting time and plasma creatinine. Additional co-factors in each of the remaining models are indicated in the figure. (**e**) Similar to (**d**) but with a tenfold increase in sample size. Multiple linear regression analyses with plasma proglucagon as the dependent variable and T2D and BMI as independent variables. Increments are given as SDs of BMI. Model 1 for each variable included adjustment for age, sex, fasting time and plasma creatinine. Additional co-factors in each of the remaining models are indicated in the figure. T2D, type 2 diabetes

Plasma proglucagon was available as a part of the proteomics analysis in a sub-cohort that matched the UK Biobank cohort on age, sex, and recruitment centre [10]. BMI, type 2 diabetes prevalence, liver fat, and HbA_1c_ were also similar between the two cohorts (ESM Table 5). We investigated whether plasma proglucagon (available as NPX units [an arbitrary and normalised unit on a log_2_ scale]) was associated with BMI, type 2 diabetes and MASLD. Plasma proglucagon was increased in individuals with obesity (fold change [FC] 1.43, *p*=1×10^−72^) (Fig. 1a), type 2 diabetes (FC 2.36, *p*=3×10^−75^) (Fig. 1b) and MASLD (FC 1.23, *p*=1×10^−6^) (Fig. 1c).

We performed multiple linear regression models to assess the association between plasma proglucagon as independent variable and type 2 diabetes, BMI and liver fat as dependent variables. Model 1 was adjusted for age, sex, fasting time and plasma creatinine. All variables associated with higher plasma proglucagon (Fig. 1d). To investigate whether these metabolic diseases were independently associated with higher proglucagon, we adjusted the linear models for each of the other variables. Plasma proglucagon remained positively associated with BMI (*p*=0.0004), type 2 diabetes (*p*=1.4×10^−6^) and liver fat (*p*=0.0027) (Fig. 1d), suggesting that each of these metabolic disorders independently associate with elevations in plasma proglucagon. This was confirmed in an additional analysis with a tenfold increase in the sample size for type 2 diabetes and BMI specifically (Fig. 1e). Individuals with type 2 diabetes on metformin treatment had higher proglucagon levels, whereas insulin treatment lowered proglucagon to levels below the reference levels of individuals without diabetes (ESM Fig. 1).

**Fig. 2:**
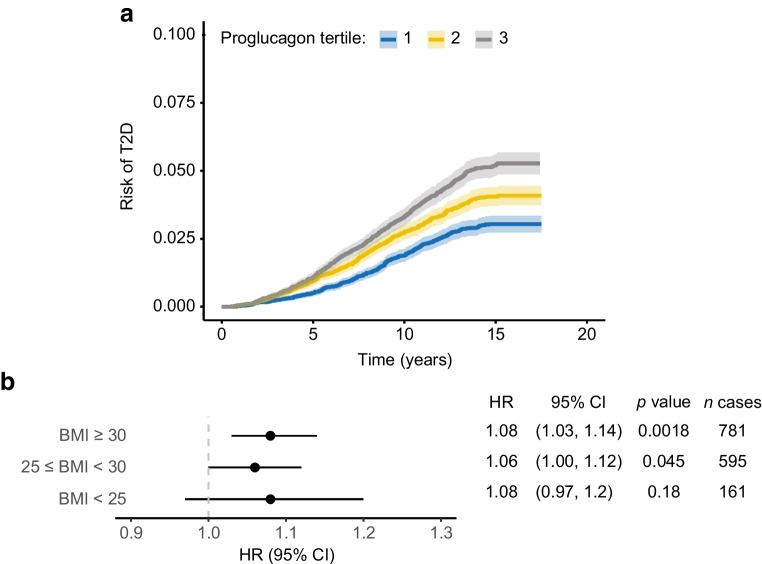
(**a**) Kaplan-Meiner survival curves for incident type 2 diabetes during the follow-up period, differentiated by tertiles of baseline proglucagon levels. The shaded areas represent 95% CI. Tertile 1: *n*=13,385, mean proglucagon=−1.50 NPX; Tertile 2: *n*=13,386, mean proglucagon=−0.005 NPX; Tertile 3: *n*=13,385, mean proglucagon=1.62 NPX. The subgroups were statistically compared using pairwise logrank test with Bonferroni correction. (**b**) Results from Cox proportional hazard regression analysis. The model was stratified on BMI categories: normal-weight, BMI <25 kg/m^2^; overweight, 25 kg/m^2^ ≤ BMI <30 kg/m^2^; obese, BMI ≥30 kg/m^2^. Proglucagon (NPX) was the independent variable and age (5 year increment) and sex were included as covariates

To investigate if high plasma levels of proglucagon associate with an increased risk of developing type 2 diabetes, we first performed a Kaplan–Meier survival analysis stratified on tertiles of plasma proglucagon at baseline (Tertile 1: mean proglucagon (NPX): −1.50, *n*=13,385; Tertile 2: mean proglucagon: −0.005, *n*=13,386; Tertile 3: mean proglucagon: 1.62, *n*=13,385). The median follow-up time was 14.75 years, and the number of incident type 2 diabetes cases included in the models was 1551 (893 men and 658 women). The risk of incident type 2 diabetes increased stepwise from low to medium levels (*p*=1.5×10^−5^) and from medium to high levels (*p*=2.4×10^−5^) (Fig. 2a). Second, we applied a Cox proportional hazard regression analysis to evaluate the impact of baseline proglucagon levels of incident type 2 diabetes with adjustment for age and sex. Proglucagon was positively associated with the risk of type 2 diabetes development (HR 1.13; 95% CI 1.09, 1.17, *p*=1.3×10^−12^). We also stratified our model on BMI, and the association between proglucagon and incident type 2 diabetes was of similar magnitude across BMI categories (Fig. 2b).

### Association between plasma proglucagon and circulating metabolites

**Fig. 3:**
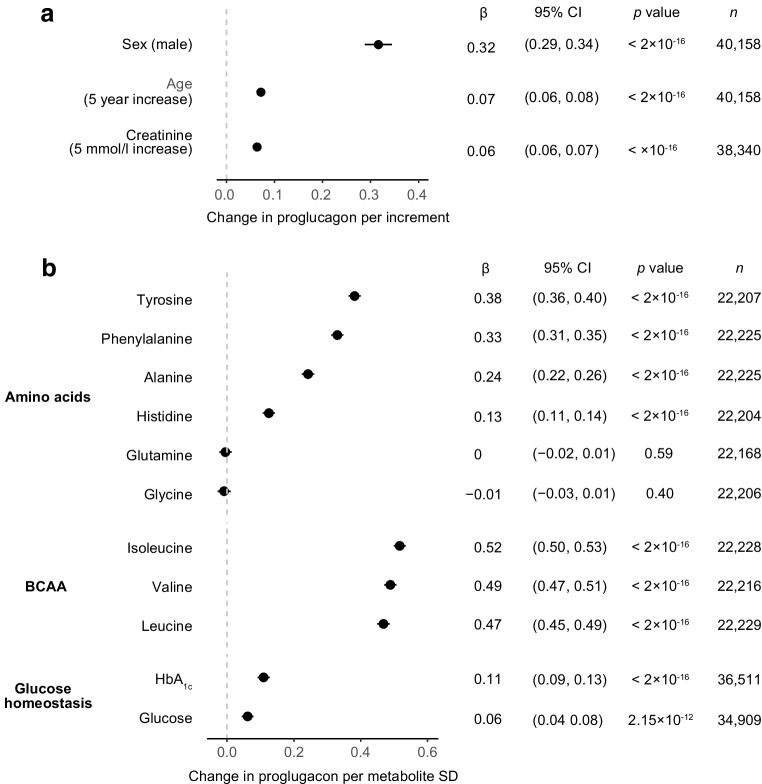
Effects of circulating metabolites on plasma proglucagon levels. (**a**) Simple linear regression analyses were performed with plasma proglucagon in NPX units as the dependent variable and the indicated confounders as independent variables. Increments were set to 5 years for age and 5 mmol/l for creatinine. (**b**) Multiple linear regression analyses were adjusted for BMI, sex, age, creatinine and fasting time. The *x*-axis is the effect size in SDs of the metabolite. *p* values were adjusted using false discovery rate (FDR) correction

We tested the impact of selected confounders on plasma levels of proglucagon using linear models. Proglucagon levels were higher in male than female participants and increased with age (Fig. 3a). Plasma creatinine served as a renal function estimate, as circulating products of proglucagon are cleared in the kidneys. Increased plasma creatinine, suggestive of reduced renal clearance, was associated with increased proglucagon (Fig. 3a).

Next, we used multiple linear regression to evaluate the association between plasma proglucagon and amino acids and glucose, adjusting for BMI and the confounders in Fig. 3a. Plasma proglucagon was positively associated with tyrosine, phenylalanine, alanine and histidine as well as the branched-chain amino acids (BCAA) valine, leucine and isoleucine. Interestingly, glutamine and glycine were not associated with proglucagon levels (Fig. 3b). Plasma glucose and HbA_1c_ were also positively associated with proglucagon levels (Fig. 3b).

### Associations of proglucagon with type 2 diabetes and BMI may depend on genetic variation of the glucagon receptor

**Fig. 4:**
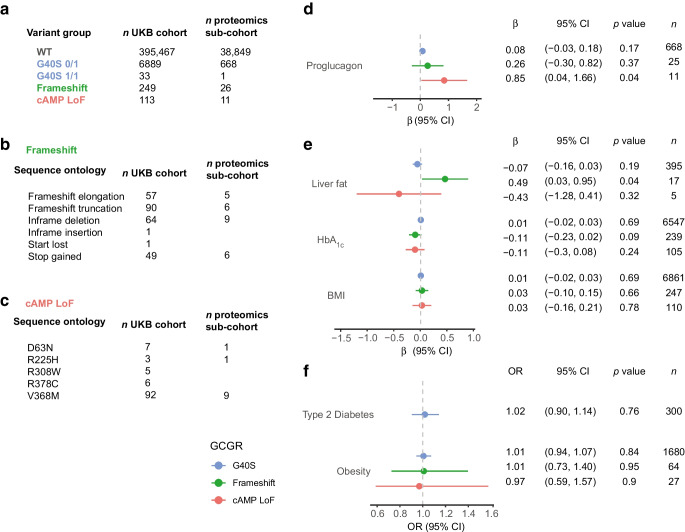
Association of LoF variants with plasma proglucagon and metabolic traits. (**a**) Number of individuals within each glucagon receptor variant group in the UK Biobank cohort and the proteomics sub-cohort. Heterozygous, 0/1; homozygous, 1/1. (**b**) The Frameshift variant group divided into the sequence ontology terms included in the group. (**c**) The cAMP LoF variant group divided according to the missense mutations included in the group. (**d**) Variant groups were tested for association with plasma proglucagon in a multiple linear model adjusted for age, sex, BMI, fasting time and plasma creatinine. G40S heterozygous and homozygous were pooled. (**e**) Variant groups were tested for associations with quantitative traits in multiple linear models adjusted for age and sex. For liver fat, the *x*-axis is the effect size (β) in 5% increments. For HbA_1c_ and BMI, β is given in SDs of the phenotype. (**f**) Variant groups were tested for association to binary traits with logistic regression with Firth correction adjusted for age and sex. Obesity was defined as BMI ≥30 and controls as BMI <25. *n* indicates the number of individuals in each model (**d**, **e**) and the number of cases within each variant group (**f**). G40S was included as a numeric predictor in the linear and logistic models (1, heterozygous; 2, homozygous). UKB, UK Biobank; WT, wild-type

To investigate the effect of genetic variation in the glucagon receptor on the same metabolic outcomes as above, we identified individuals with genetic variants of the glucagon receptor from whole-exome sequencing data. We grouped the glucagon receptor variants the following way: (1) the SNP G40S, previously associated with non-insulin-dependent diabetes, hypertension and adiposity [21–23], but normal cAMP signalling and reduced β-arrestin signalling [5], (2) the missense variants V368M, R378C, R225H, R308W and D63N were grouped as ‘cAMP LoF’ based on previous research [5], and (3) variants annotated as frameshift or stop-codon gained were grouped as ‘Frameshift’ variants (ESM Table 2). Figure 4a–c outlines the number of individuals in each group in the UK Biobank cohort and the sub-cohort included in the proteomics analysis.

Although only 11 individuals heterozygous for a cAMP LoF variant were included, we observed an elevation in proglucagon compared with the wild-type reference group (β 0.85; 95% CI 0.04, 1.66; *p*=0.04) (Fig. 4d). G40S or frameshift variants were not associated with proglucagon levels, implying that cAMP rather than β-arrestin signalling may be involved in metabolic processes directly or indirectly regulating proglucagon levels.

In the frameshift variant group including 17 individuals, liver fat was increased compared with the wild-type reference group (β 0.49; 95% CI 0.03, 0.95; *p*=0.04) (Fig. 4e). None of the glucagon receptor variant groups were associated with BMI as a continuous or binary trait. We observed no difference in the prevalence of type 2 diabetes between individuals carrying G40S and the wild-type reference group (Fig. 4f). The sample size of type 2 diabetes was inadequate for cAMP LoF and Frameshift variant groups.

To test if the association between plasma proglucagon and type 2 diabetes and BMI, respectively, was dependent on the glucagon receptor genotype, we performed logistic and linear models stratified on the variant groups with inclusion of the interaction term proglucagon×genotype. Interestingly, although plasma proglucagon was associated with type 2 diabetes in the whole cohort (Fig. 1d,e), this was not the case in individuals with G40S (*p*=0.1) (Table 1). The interaction term between G40S and proglucagon was *p*=0.073. The effect of plasma proglucagon on BMI was larger in carriers of the G40S variants compared with carriers of the wild-variant (β 0.68; 95% CI 0.43, 0.93 vs β 0.33; 95% CI 0.29, 0.36; *p*=0.013) (Table 1). The effect of plasma proglucagon on BMI in carriers of cAMP LoF and frameshift variants were not different from individuals with wild-type receptors; however, the results are limited by the low sample size (11 and 24, respectively) (Table 1).

**Table 1:** Effect of interaction between plasma proglucagon and genotype on type 2 diabetes and BMI

Genotype	*n*	Effect of plasma proglucagon (Model 1)			Interaction between plasma proglucagon and genotype (Model 2)
		β	95% CI	*p* value	*p* value
Type 2 diabetes					
WT	36,819 (control) 776 (T2D)	0.46	(0.43, 0.5)	<0.0001	
G40S	630 (control) 39 (T2D)	0.21	(−0.04, 0.46)	0.107	0.073
BMI					
WT	38,662	0.33	(0.29, 0.36)	<0.0001	
G40S	669	0.68	(0.43, 0.93)	<0.0001	0.013
cAMP LoF	11	2.98	(−4.65, 10.62)	0.339	0.141
Frameshift	24	−0.15	(−1.85, 1.55)	0.853	0.389

For type 2 diabetes, model 1 was a logistic model with type 2 diabetes as the dependent variable and proglucagon as independent variable adjusted for age, sex, fasting time, creatinine and BMI, and stratified on the genotype G40S. Model 2 included the interaction between proglucagon and G40S. For BMI, model 1 was a linear model with BMI as the dependent variable and proglucagon as independent adjusted for age, sex, fasting time, creatinine and type 2 diabetes, and stratified on the glucagon receptor variant groups. Model 2 for each variant group included the interaction between proglucagon and variant group

T2D, type 2 diabetes; WT, wild-type

### Circulating amino acids and proteins may not be altered by LoF glucagon receptor variants

Plasma levels of the individual and the sum of amino acids did not show notable variations between individuals with the glucagon receptor variant groups and those with the wild-type variant (Table 2).

**Table 2:** Association between glucagon receptor variant groups and plasma amino acids

	cAMP LoF				G40S				Frameshift				SD
	β	95% CI	*p* value	*n*	β	95% CI	*p* value	*n*	β	95% CI	*p* value	*n*	
Alanine	0.20	−0.04, 0.45	0.10	63	−0.01	−0.04, 0.02	0.66	3868	−0.06	−0.22, 0.1	0.44	151	0.08
Glutamine	0.04	−0.2, 0.29	0.72	63	0.03	−0.01, 0.06	0.10	3863	0.11	−0.05, 0.26	0.18	151	0.08
Glycine	−0.20	−0.43, 0.03	0.09	63	0.02	−0.01, 0.05	0.12	3860	−0.06	−0.21, 0.09	0.42	151	0.07
Histidine	−0.03	−0.28, 0.21	0.79	63	−0.02	−0.05, 0.01	0.26	3861	0.08	−0.08, 0.24	0.33	151	0.01
Isoleucine	−0.02	−0.26, 0.22	0.88	63	0.01	−0.02, 0.04	0.55	3869	0.02	−0.13, 0.18	0.76	151	0.02
Leucine	−0.03	−0.26, 0.21	0.83	63	0.00	−0.03, 0.03	0.99	3869	0.03	−0.12, 0.18	0.68	151	0.03
Valine	−0.13	−0.36, 0.1	0.28	63	0.00	−0.03, 0.03	0.93	3866	0.01	−0.14, 0.16	0.88	151	0.04
Phenylalanine	−0.12	−0.36, 0.12	0.34	63	0.03	0, 0.06	0.08	3867	−0.06	−0.22, 0.09	0.42	151	0.01
Tyrosine	−0.03	−0.27, 0.21	0.79	63	0.02	−0.01, 0.05	0.18	3863	−0.02	−0.18, 0.13	0.77	151	0.02
Total AA	−0.01	−0.26, 0.23	0.92	63	0.02	−0.01, 0.05	0.27	3845	0.01	−0.15, 0.17	0.94	151	0.21

The associations were tested in linear models with the genotype as predictor and age, sex, BMI and fasting time as covariates. β (effect size) is given in amino acid SD

AA, amino acids

We next performed differential expression analysis on the proteomics dataset (~1500 proteins measured in each sample) to identify plasma proteins potentially regulated by glucagon receptor signalling. After correction for multiple testing, no proteins reached statistical significance. A list of the top ten up- and downregulated proteins for each variant group is provided in Table 3.

**Table 3:** Top ten up- and downregulated proteins in glucagon receptor variant groups

	cAMP LoF				G40S				Frameshift			
	Protein	Log_2_FC	*p* value	adj. *p* value	Protein	Log_2_FC	*p* value	adj. *p* value	Protein	Log_2_FC	*p* value	adj. *p* value
Upregulated	IL18RAP	0.644	0.225	0.916	GRAP2	0.156	0.004	0.384	RUVBL1	0.852	2.2×10^−4^	0.06
	PNLIPRP2	0.639	0.478	0.916	MESD	0.155	0.003	0.384	IL5	0.661	0.026	0.779
	NTPROBNP	0.617	0.058	0.916	STAT5B	0.15	0.01	0.44	PAEP	0.512	0.065	0.988
	KLK1	0.568	0.156	0.916	RHOC	0.144	0.004	0.384	TSHB	0.44	0.008	0.487
	GCG	0.513	0.194	0.916	LAT2	0.129	0.015	0.44	PM20D1	0.417	0.27	0.999
	AK1	0.468	0.026	0.916	PDLIM7	0.127	0.019	0.44	PNLIPRP2	0.378	0.001	0.191
	GHRL	0.459	0.082	0.916	DIABLO	0.126	0.004	0.384	DSG4	0.378	0.531	0.999
	NPPB	0.457	0.269	0.916	GOPC	0.126	0.005	0.384	NPM1	0.364	0.052	0.897
	SESTD1	0.431	0.05	0.916	NCK2	0.126	0.007	0.384	SPINK4	0.343	0.015	0.712
	CHIT1	0.43	0.357	0.916	SULT1A1	0.124	0.023	0.449	KLB	0.342	0.037	0.875
Downregulated	MICB_MICA	−0.97	0.03	0.92	IL18RAP	−0.11	0.11	0.79	FABP1	−0.49	0.02	0.72
	SIGLEC5	−0.86	0.02	0.92	KIR3DL1	−0.11	0.03	0.45	OXT	−0.49	0.12	0.99
	GPA33	−0.77	0.07	0.92	FOLR3	−0.10	0.22	0.80	PDGFB	−0.43	0.02	0.72
	PDCD6	−0.75	0.001	0.54	TCL1A	−0.09	0.04	0.48	FOLR3	−0.43	0.27	0.99
	FOXO3	−0.71	0.02	0.92	NPTN	−0.08	0.02	0.44	SPARC	−0.42	0.006	0.49
	CASP10	−0.67	0.005	0.73	SORD	−0.08	0.006	0.38	EPCAM	−0.40	0.05	0.90
	TDGF1	−0.58	0.20	0.92	HAO1	−0.08	0.14	0.73	GH2	−0.49	0.25	0.99
	PVALB	−0.54	0.13	0.92	GHRL	−0.08	0.03	0.44	FCGR2A	−0.39	0.003	0.49
	PTPN1	−0.52	0.05	0.92	CD177	−0.07	0.16	0.76	GUSB	−0.38	0.007	0.49
	TCL1B	−0.51	0.05	0.92	SULT2A1	−0.07	0.004	0.38	GH1	−0.37	0.32	0.99

Differential expression analysis was performed for each of the variant groups

*p* values were adjusted by FDR for multiple testing

FDR, false discovery rate

## Discussion

Using UK Biobank data encompassing multiple omics data, MRI imaging and hospital registers, we here demonstrated that increased plasma levels of proglucagon are independently associated with obesity, MASLD and type 2 diabetes. In addition, proglucagon levels were significant predictors of the risk of type 2 diabetes development over a 14-year follow-up period. Although causality cannot be established, our data support the idea that increased proglucagon directly contributes to development of type 2 diabetes [24].

One of the two antibodies in the Olink proglucagon assay binds within the first 100 amino acids of proglucagon, whereas the binding site of the other antibody is proprietary information and has not been disclosed. It is therefore unknown which section(s) of the proglucagon-derived peptide is measured by the assay, and to what extent. We previously performed a pilot in vitro analysis of human plasma spiked with 100 pmol/l of glucagon, GLP-1 7–36, GLP-1 9–36, GLP-2 and oxyntomodulin. The proglucagon assay from Olink measured both forms of GLP-1, whereas neither glucagon, GLP-2 or oxyntomodulin were detected (https://github.com/nicwin98/Olink). Antibodies raised against GLP-1 have the (dis)advantage of measuring all molecular forms containing the amino acid sequence of GLP-1. Importantly, this includes MPGF and GLP-1 1–36, both of which derive from alpha cell processing of proglucagon [25, 26]. In fact, an older study revealed that elevated levels of fasting and arginine-induced immunoreactive GLP-1 in individuals with type 2 diabetes turned out to be the pancreatic peptides MPGF and GLP-1 1–36 [27]. Thus, to interpret analyses of GLP-1 concentrations, it is essential to know if the antibodies used are N-terminal wrapping (i.e. specific for the full-length [1–36], active [7–36], or total [9–36] GLP-1), C-terminal wrapping, or binding anywhere in the middle of the peptide, in which case the assay would measure all forms as well as MPGF.

We observed that proglucagon was elevated in individuals with type 2 diabetes (Fig. 1b). Numerous studies have described that GLP-1 may be reduced, and glucagon increased, in type 2 diabetes [28, 29]. This suggest that the proglucagon assay is likely measuring a pancreatic peptide that includes the GLP-1 amino acid sequence (possibly MPGF). In line with this hypothesis, we observed an increase in plasma proglucagon in carriers of cAMP LoF variants of the glucagon receptor (Fig. 4d). Since glucagon resistance in MASLD results in a compensatory increase in glucagon levels [30], similar mechanisms may underlie the finding of increased proglucagon (alpha cell-derived) in individuals with reduced glucagon receptor cAMP signalling.

Plasma proglucagon showed a positive association with BCAA. BCAA catabolism is not regulated by glucagon, and BCAA do not directly stimulate glucagon secretion [31, 32]. However, BCAA stimulate GLP-1 secretion [33] and play an important role in the pathogenesis of insulin resistance [34, 35]. A limitation of the current study is the lack of markers of insulin resistance, such as plasma insulin levels. We previously observed that glucagon resistance and insulin resistance may coexist but importantly also occur independently of each other, highlighting the differential pathophysiological mechanisms underlying glucagon and insulin resistance [36].

In agreement with previous studies, we found a link between MASLD and glucagon. We used MRI–PDFF to diagnose MASLD, but we lacked data on the severity of fibrosis, which is the most critical predictor of clinical outcomes in MASLD. Given the probable close relationship between glucagon and the metabolic alterations in MASLD, the degree of liver fat is likely to indicate the risk of developing cardiometabolic conditions, including diabetes. Our findings therefore support speculations that there is a two-way connection between MASLD and diabetes and suggest that glucagon could be a main factor in the pathogenesis.

Consistent with prior research, we identified an association between plasma proglucagon and prevalent type 2 diabetes. Metformin treatment is known to increase GLP-1 and reduce glucagon secretion, thus, the increase in proglucagon by metformin in individuals with type 2 diabetes may represent increased plasma GLP-1 levels. Conversely, insulin is an effective inhibitor of glucagon secretion from alpha cells, and the markedly lower levels of proglucagon in individuals on insulin is most likely a consequence of lower glucagon levels. Collectively, these data support the hypothesis that the proglucagon assay measures a combination of intestinal and pancreatic proglucagon products (probably GLP-1 and MPGF, respectively).

Type 2 diabetes is often diagnosed by general practitioners, and only ~41% of diabetes diagnoses are registered in hospital records [17]. Primary care data are linked to the UK Biobank for ~45% of the participants up until 2016 (England) and 2017 (Scotland and Wales), so to get a longer follow-up period, incident type 2 diabetes was defined from secondary care ICD-10 diagnostic codes. This is a limitation of this study and may explain the flattening of the Kaplan–Meier curve (Fig. 2a) in the later years of the follow-up period, as hospital diagnoses may be registered at a later point than the primary care diagnosis. Furthermore, UK Biobank is not representative of the UK population with evidence of a selection bias towards more healthy volunteers regarding obesity, smoking, and alcohol consumption [37]. There are more female than male participants in the UK Biobank, and to address potential confounding effects related to sex differences, we have included sex as a covariate in all our analyses. Another limitation is that quantification of liver fat is obtained from the MRI scan performed ~10.5 years after the baseline data was obtained.

Traditionally, the primary focus on glucagon receptor signalling has centred on the adenylate cyclase/cAMP/protein kinase A pathway as the predominant mediator of the hepatic glucose-mobilising actions of glucagon. However, compelling evidence suggests that the PLC/IP3 pathway may be even more important for physiological levels of glucagon, compared with pathologically or pharmacologically elevated levels [38]. Further investigation into the impact of rare missense variants on this pathway is warranted.

The most common missense variant in the glucagon receptor, G40S, has normal to mildly reduced cAMP signalling [39–41], and substantially decreased β-arrestin signalling [5]. G40S has previously been linked to non-insulin-dependent diabetes and central adiposity in France and Sardinia [21, 22], but interestingly not in Japan or Finland [42, 43]. We did not find an increased prevalence of type 2 diabetes or obesity in heterozygotes or homozygotes of the G40S variant in the UK Biobank. However, there appeared to be an interaction between plasma proglucagon and G40S, with proglucagon having a stronger association with BMI in carriers of G40S compared with carriers of the wild-type variant (Table 1). In contrast, the association between proglucagon and type 2 diabetes was weaker in individuals with G40S compared with the wild-type variant (*p*=0.107, Table 1). Together with the divergent literature on the effects of G40S on metabolic disorders, our results suggest that G40S may play a role in the development of type 2 diabetes and obesity, but that this may require parallel metabolic disruptions leading to altered proglucagon levels.

Previous research has indicated a potential association between cAMP LoF variants and an increased risk of obesity [5]. Since then, exome sequencing data was increased to cover the whole UK Biobank. After an approximate doubling of the sample size of carriers of a cAMP LoF variant, this tendency disappeared, both when BMI was treated as a continuous and dichotomised variable. However, the increased plasma levels of proglucagon in this group of individuals suggest that cAMP signalling is involved in the regulation of a factor, that in turn regulates proglucagon levels in a feedback manner. This has previously been suggested to be amino acids, with alanine being particularly important for this feedback system, termed the liver–alpha cell axis [44–46]. In line with this, there was a tendency to increased alanine (*p*=0.1) in individuals with cAMP variants adjusting for age, sex, BMI, fasting time and creatinine. Because the cAMP LoF variants are very rare, we aimed at creating the most homogenous study population by excluding participants who were not categorised as white, and participants with sex chromosome aneuploidy. This lack of diversity may restrict the generalisability of our findings to other ethnic or racial groups.

The Frameshift category of glucagon receptors was defined rather broadly. Yet, carriers of one of the Frameshift variants were associated with higher levels of liver fat compared with carriers of the wild-type variant. This was not observed in G40S or the cAMP LoF variant groups, suggesting that a signalling pathway other than cAMP and β-arrestin is likely involved. Further functional subdivision and in silico predictions may help select and group variants that are pharmacologically characterised by more stratified LoF phenotypes. Other research groups have found ubiquitination and β-arrestin to be essential for the signalling and internalisation of the glucagon receptor [4, 47], whereas others report only a minor internalisation of the glucagon receptor [48], suggesting that this pathway may not be a major regulator of glucagon receptor signalling. The multiple factors impacting signalling pathways are complex and warrant further exploration.

In conclusion, our study supports the involvement of glucagon signalling in metabolic disorders such as type 2 diabetes and MASLD and that increased proglucagon levels may predispose to type 2 diabetes. Furthermore, the presence of hyperglucagonaemia in obesity, MASLD and type 2 diabetes indicates that distinct mechanisms may drive increased alpha cell secretion. Signalling pathways other than cAMP and β-arrestin recruitment may be important for the metabolic effects of glucagon such as the regulation of liver fat. Identification of the specific molecular characteristics responsible for the beneficial effects of glucagon on hepatic lipid turnover may be crucial in developing improved glucagon co-agonists for the treatment of MASLD and obesity.

### Supplementary Information

Below is the link to the electronic supplementary material.

Supplementary file1 (PDF 273 KB)

## Data Availability

All data used in this work can be acquired from the UK Biobank (https://www.ukbiobank.ac.uk/). All coding is available through https://github.com/nicwin98/UK-Biobank-GCG. Nomenclature used by Olink, matching the UniProt database, can be downloaded from https://biobank.ndph.ox.ac.uk/ukb/coding.cgi?id=143.

## Abbreviations

BCAA: Branched-chain amino acid(s)
FC: Fold change
GLP-1: Glucagon-like peptide-1
gVCF: Genomic variant call format
IP3: Inositol phosphate
LoF: Loss-of-function
MASLD: Metabolic dysfunction-associated steatotic liver disease
MPGF: Major proglucagon fragment
NPX: Normalised Protein eXpression
PDFF: Proton density fat-fraction
PLC: Phospholipase C
