# Supplementary material for: Determinants of plasma levels of proglucagon and the metabolic impact of glucagon receptor signalling: a UK Biobank study
Source: Diabetologia. 2024 May 6;67(8):1602–15. doi: 10.1007/s00125-024-06160-1 (PMC11343844; doi:10.1007/s00125-024-06160-1)
Supplement: Supplementary file 1 — Supplementary file1 (PDF 273 KB) [file 125_2024_6160_MOESM1_ESM.pdf]

Electronic Supplementary Material (ESM)

Determinants of plasma levels of proglucagon and the metabolic impact of glucagon receptor signalling: a UK Biobank study

Marie Winther-Sørensen, Sara L. Garcia, Andreas Bartholdy, Maud Eline Ottenheijm, Karina Banasik, Søren Brunak, Charlotte M. Sørensen, Lise Lotte Gluud, Filip K. Knop, Jens Juul Holst, Mette M. Rosenkilde, Majken K. Jensen, Nicolai J. Wewer Albrechtsen

Diabetologia (2024)

ESM Table 1

UK Biobank covariates used in the study.

**Covariables**	**Definitions**	**UKB data field**
**Age**	Age when attended the assessment center at the initial assessment, truncated to a full year.	21003
**Sex**	Acquired from central registry at recruitment.	31
**Race**	Categorical variable, white and non-white. Indicates samples who self-identified as 'White British' at the initial touchscreen questionnaire and have very similar genetic ancestry based on a principal components analysis of the genotypes.	22006
**Body mass index (BMI)**	Weight/height^2, kg/m^2. BMI when attended the assessment center at the initial assessment.	21001
**Liver fat**	%. Proton density fat fraction measured by MRI scan at instance 2.	40061
**Date attending assessment center**	Date of attending assessment centre.	53
**Date of death**	Date of death. Acquired from central registry.	40000
**Date lost to follow up**	Date lost to follow-up. This data was last updated in May 2017.	191

**Sex chromosome aneuploidy**	Samples which were identified as putatively carrying sex chromosome configurations that are not either XX or XY	22019
**HbA_{1c}**	Baseline HbA_{1c} levels, mmol/mol	30750
**Glucose**	Baseline glucose measurement, mmol/l	30740
**Weekly alcohol consumption**	Calculated as the sum of beer, cider, champagne, white wine, red wine, fortified wine, spirits, and other alcoholic drinks.	1588, 1578, 1608, 5364, 1568, 1598
**Amino acids**	Plasma levels of amino acids measured by NMR metabolomics	23460, 23461, 23462, 23463, 23465, 23466, 23467
**Proteomics**	Only available through the Research Analysis Platform. A list of field names is available at https://github.com/nicwin98/UK-Biobank-GCG	
**Medication**	A list of diabetes medications is provided in ESM table 4.	20003

ESM Table 2

Mutations in the glucagon receptor included in the Frameshift variants group

Frameshift mutations		
Chrom_pos_ref_alt	*n*	Sequence Ontology
chr17_81809019_A_G	1	start lost
chr17_81809021_GC_G	2	frameshift truncation
chr17_81809027_CT_C	1	frameshift truncation
chr17_81809039_G_GCGACC	1	frameshift elongation
chr17_81809040_C_T	5	stop gained
chr17_81809045_CCT_C	22	frameshift truncation
chr17_81809064_C_CTGCT	33	frameshift elongation
chr17_81809838_C_A	1	stop gained
chr17_81810872_AC_A	1	frameshift truncation
chr17_81810888_CG_C	3	frameshift truncation
chr17_81810897_TCTC_T	5	inframe deletion
chr17_81810907_CT_C	40	frameshift truncation
chr17_81811122_TG_T	1	frameshift truncation
chr17_81811273_T_TACA	1	inframe insertion
chr17_81811283_CCCTGGGGGCCCTGCTCCTCGCCTTGGCCAT_C	12	inframe deletion
chr17_81811286_TG_T	3	frameshift truncation
chr17_81811293_C_GT	1	frameshift elongation
chr17_81811316_TG_T	1	frameshift truncation
chr17_81811316_T_TG	14	frameshift elongation
chr17_81811407_G_GCTGCA	2	frameshift elongation
chr17_81811422_CA_C	1	frameshift truncation
chr17_81811441_CTGTT_C	1	frameshift truncation
chr17_81811710_C_A	6	stop gained
chr17_81811751_T_TG	1	frameshift elongation
chr17_81811887_TGCCCC_T	3	frameshift truncation
chr17_81811911_CT_C	1	frameshift truncation
chr17_81811923_CAAGT_C	2	frameshift truncation
chr17_81812181_AG_A	1	frameshift truncation
chr17_81812216_G_A	8	stop gained
chr17_81812248_TC_T	1	frameshift truncation

chr17_81812579_C_CA	5	frameshift elongation
chr17_81812581_ACTT_A	47	inframe deletion
chr17_81812598_CG_C	1	frameshift truncation
chr17_81812607_C_T	2	stop gained
chr17_81812838_CT_C	1	frameshift truncation
chr17_81812929_TC_T	1	frameshift truncation
chr17_81813477_C_T	1	stop gained
chr17_81813509_G_A	3	stop gained
chr17_81813529_G_A	8	stop gained
chr17_81813609_AG_A	2	frameshift truncation
chr17_81813624_C_T	15	stop gained
chr17_81813647_CT_C	1	frameshift truncation

ESM Table 3

Definition of diseases

Disease	Definitions	UKB data field
**Baseline type 2 diabetes**	Categorical variable. Cases were defined as probable and possible type 2 diabetes, and controls were defined as unlikely diabetes based on the Eastwood algorithm.	
**Incident type 2 diabetes**	Categorical variable. First, T2D was defined using ICD-10 codes for diabetes: E11 (“type 2 diabetes mellitus”) and E14 (”unspecified diabetes mellitus”) and the dates for the diagnosis (*n* = 3585). To refine the categorization further, the following steps were implemented:<br>1. Individuals additionally diagnosed with E10 (“type 1 diabetes mellitus”) were removed from T2D (*n* = 360).<br>2. Individuals with probably and possibly type 2 diabetes based on the Estwood algorithm were removed from T2D (*n* = 1370).<br>3. Individuals with ICD10 code of T2D given prior to the baseline were removed from T2D (*n* = 304).<br>4. Individuals with baseline HbA1c > 48 mmol/mol were removed from T2D (*n* = 159).<br>This classification process resulted in 1,562 incident cases of T2D (893 men, 658 women).	130708<br>130714<br>41270<br>30750
**MASLD**	Categorical variable. Cases were defined as > 5.5% fat on MRI PDFF and an alcohol consumption < 30g/day for men and < 20g/day for women. Individuals with MRI PDFF > 5.5% and an alcohol consumption > 30g/day for men and > 20g/day for women were excluded from the control group.	40061, 1588, 1578, 1608, 5364, 1568, 1598
**Obesity**	Categorical variable. Cases were defined as BMI > 30 and controls as BMI < 25.	21001

ESM Table 4

List of medication codes for type 2 diabetes drugs (data field 20003, baseline visit). The list can be downloaded from the UK Biobank website https://biobank.ctsu.ox.ac.uk/crystal/coding.cgi?id=4.

**Medication**	**Medication code**
Metformin	1140884600, 1140874686, 1141189090
Insulin	1140883066
Sulfonylureas	1140874718, 1140874744, 1140874746, 1141152590, 1141156984, 1140874646, 1141157284, 1140874652, 1140874674, 1140874728
Other (acerbose, glucotard)	1140868902, 1140868908, 1140857508
Meglitinides	1141173882, 1141173786, 1141168660
Glitazones	1141171646, 1141171652, 1141153254, 1141177600, 1141177606
Non-metformin OADs	The combination of sulfonylureas, other, meglitinides, and glitazones

ESM Table 5

Baseline characteristics of the UK Biobank cohort and the sub-cohort included in the proteomics analysis. Prevalent type 2 diabetes (T2D) was defined from the Eastwood algorithm [1].

**Characteristic**	**UKB cohort**	**Proteomics sub-cohort**
N	408,931	40,158
Male sex	187,863 (45.9)	18,585 (46.3)
Age	56.9 (8)	57.2 (8.1)
BMI (kg/m^2) [a]	27.4 (4.8)	27.4 (4.7)
Prevalent T2D	17,719 (4.3)	1842 (4.6)
Liver fat (%)*[a]	4.8 (4.9)	4.8 (4.8)
HbA_{1c} (mmol/mol) [a]	36 (6.5)	36.1 (6.7)

Continuous variables are presented as mean (SD) and categorical variables as n (%)

*Liver fat was quantified as MRI-PDFF at the second repeat (imaging) visit (median time from enrolment visit: 10.5 years).

[a] Missing data were present for BMI: a) *n*=1294 (0.3%), b) *n*=160 (0.4%); Liver fat: a) 384,507 (91.6%), b) 35,910 (89,4%); HbA_{1c}: a) *n*=19,135 (4.7%), b) 1812 (4.5%).

ESM Figure 1

Effect of type 2 diabetes drugs on the association between type 2 diabetes and plasma proglucagon.

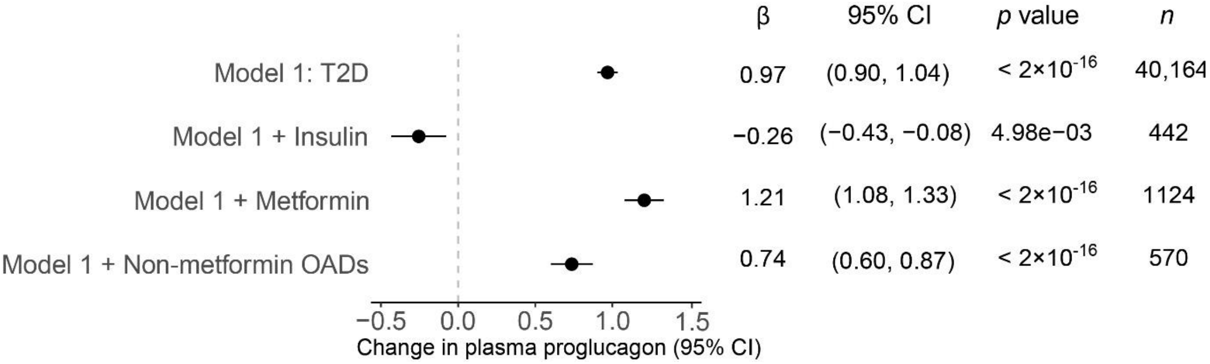

Multiple linear regression analyses with plasma proglucagon as the dependent variable and T2D as the independent variable. Model 1 was adjusted for age, sex, fasting time, and plasma creatinine. The additional co-factor in each of the remaining models are indicated in the figure (insulin, metformin, and non-metformin oral anti-diabetic drugs (OAD). CI, 95% confidence intervals; T2D, type 2 diabetes.

References

1. Eastwood, S.V., et al., *Algorithms for the Capture and Adjudication of Prevalent and Incident Diabetes in UK Biobank.* PLOS ONE, 2016. **11**(9): p. e0162388.
